# Supplementary material for: Tackling Food Waste: Impact of German Consumer Behaviour on Food in Chilled Storage
Source: Foods. 2020 Oct 14;9(10):1462. doi: 10.3390/foods9101462 (PMC7602148; doi:10.3390/foods9101462)
Supplement: Supplementary file 1 [file foods-09-01462-s001.pdf]

Table 1: Raw data, characteristics of respondents and their refrigerator appliances

Gender

		respondents	percentages	valid percentages	accumulated percentages
valid	male	1347	50.5	51%	50.5
	female	1319	49.5	49%	100
	sum	2666	100	100	

Age Group

		respondents	percentages	valid percentages	accumulated percentages
valid	18-29	417	15.6	15.6	15.6
	30.39	678	25.4	25.4	41.1
	40-49	500	18.8	18.8	59.8
	50-59	596	22.4	22.4	82.2
	60-69	475	17.8	17.8	100
	sum	2666	100	100	

Household Size

		respondents	percentages	valid percentages	accumulated percentages
valid	1	1276	47.9	48%	47.9
	2	837	31.4	31%	79.3
	3	281	10.5	11%	89.8
	4	203	7.6	8%	97.4
	>4	69	2.6	3%	100
	sum	2666	100	100	

Responsibility for food management

		respondents	percentages	valid percentages	accumulated percentages
valid	fully	2027	76	76%	76
	someone else	107	4	4%	80
	jointly	532	20	20%	100
	sum	2666	100	100	

Age of Main refrigerator

		respondents	percentages	valid percentages	accumulated percentages
valid	< 2 yrs	487	18.3	18.30%	18.3
	2 - 5 yrs	932	35	35.00%	53.2
	6 - 10 yrs	607	22.8	22.80%	76
	> 10 yrs	540	20.3	20.30%	96.2
	do not know	100	3.8	3.80%	100
	sum	2666	100	100	

Number of Refrigerators

		respondents	percentages	valid percentages	accumulated percentages
valid	1	1492	56	56%	56
	≥ 2	1174	44	44%	100
	sum	2666	100	100	

Table 2: Raw data, load of refrigerator

Load of Refrigerator Compertment

		respondents	percentages	valid percentages	accumulated percentages
valid	maximum	708	26.6	26.60%	26.6
	average	1537	57.7	57.70%	84.2
	minumum	409	15.3	15.30%	99.5
	no load	12	0.5	0.50%	100
	sum	2666	100	100	

Load of Vegetable Drawer

		respondents	percentages	valid percentages	accumulated percentages
valid	maximum	629	23.6	23.60%	23.6
	average	1272	47.7	47.70%	71.3
	minumum	624	23.4	23.40%	94.7
	no load	141	5.3	5.30%	100
	sum	2666	100	100	

Table 3: Raw data, storage times reported in days for of highly perishable foods

Fruit

		respondents	percentages	valid percentages	accumulated percentages
valid	1 day	81	3	3.2	3.2
	3 days	815	30.6	32.2	35.4
	5 days	856	32.1	33.9	69.3
	7 days	599	22.5	23.7	93
	14 days	132	5	5.2	98.2
	> 14 days	45	1.7	1.8	100
	sum	2528	94.8	100	
not valid		138	5.2		
sum		2666	100		

Vegetables

		respondents	percentages	valid percentages	accumulated percentages
valid	1 day	60	2.3	2.3	2.3
	3 days	843	31.6	32.6	34.9
	5 days	887	33.3	34.3	69.3
	7 days	591	22.2	22.9	92.1
	14 days	160	6	6.2	98.3
	> 14 days	43	1.6	1.7	100
	sum	2584	96.9	100	
not valid		82	3.1		
sum		2666	100		

Meat

		respondents	percentages	valid percentages	accumulated percentages
valid	1 day	340	12.8	13.7	13.7
	3 days	1155	43.3	46.5	60.2
	5 days	532	20	21.4	81.6
	7 days	320	12	12.9	94.5
	14 days	68	2.6	2.7	97.2

	> 14 days	69	2.6	2.8	100
	sum	2484	93.2	100	
not valid		182	6.8		
sum		2666	100		

Fish					
		respondents	percentages	valid percentages	accumulated percentages
valid	1 day	814	30.5	37.4	37.4
	3 days	905	33.9	41.6	79
	5 days	204	7.7	9.4	88.4
	7 days	123	4.6	5.7	94
	14 days	47	1.8	2.2	96.2
	> 14 days	83	3.1	3.8	100
	sum	2176	81.6	100	
not valid		490	18.4		
sum		2666	100		

Table 4: Raw data, storage places of highly perishable foods

Fruit					
		respondents	percentages	valid percentages	accumulated percentages
valid	upper shelf	88	3.3	3.5	3.5
	mid shelf	200	7.5	7.9	11.4
	bottom shelf	275	10.3	10.9	22.3
	door rack	29	1.1	1.1	23.4
	Vegetable Dra	1085	40.7	42.9	66.3
	Other Drawer	84	3.2	3.3	69.7
	Outside	767	28.8	30.3	100
	sum	2528	94.8	100	
not valid		138	5.2		
sum		2666	100		

Vegetables					
		respondents	percentages	valid percentages	accumulated percentages
valid	upper shelf	37	1.4	1.4	1.4
	mid shelf	142	5.3	5.5	6.9
	bottom shelf	139	5.2	5.4	12.3
	door rack	17	0.6	0.7	13
	Vegetable Dra	2101	78.8	81.3	94.3
	Other Drawer	32	1.2	1.2	95.5
	Outside	116	4.4	4.5	100
	sum	2584	96.9	100	
not valid		82	3.1		
sum		2666	100		

Meat					
		respondents	percentages	valid percentages	accumulated percentages
valid	upper shelf	378	14.2	15.2	15.2
	mid shelf	1053	39.5	42.4	57.6
	bottom shelf	797	29.9	32.1	89.7
	door rack	22	0.8	0.9	90.6

	Vegetable Dra	45	1.7	1.8	92.4
	Other Drawer	117	4.4	4.7	97.1
	Outside	72	2.7	2.9	100
	sum	2484	93.2	100	
not valid		182	6.8		
sum		2666	100		

Fish					
		respondents	percentages	valid percentages	accumulated percentages
valid	upper shelf	346	13	15.9	15.9
	mid shelf	760	28.5	34.9	50.8
	bottom shelf	705	26.4	32.4	83.2
	door rack	26	1	1.2	84.4
	Vegetable Dra	40	1.5	1.8	86.3
	Other Drawer	118	4.4	5.4	91.7
	Outside	181	6.8	8.3	100
	sum	2176	81.6	100	
not valid		490	18.4		
sum		2666	100		

Table 5: Raw data, correlations between demographic characteristics (age groups) and the rate of food waste for food categories

Food Waste - Age Groups						
**Correlations**						
			Food Waste Rate - Fruit	Food Waste Rate - Vegetables	Food Waste Rate - Meat	Food Waste Rate - Fish
Spearman-Rho	Agegroups	Correleation Coefficient	,263**	,289**	,256**	,232**
		sig. (2-sided)	0.000	0.000	0.000	0.000
		N	2528	2584	2484	2176

Table 6: Raw data, correlations between demographic characteristics (household size) and the rate of food waste for food categories

Food Waste - Household Size						
**Correlations**						
			Food Waste Rate - Fruit	Food Waste Rate - Vegetables	Food Waste Rate - Meat	Food Waste Rate - Fish
Spearman-Rho	Househild size	Correleation Coefficient	-,140**	-,135**	-,123**	-,121**
		sig. (2-sided)	0.000	0.000	0.000	0.000
		N	2528	2584	2484	2176

Table 7: : Raw data, correlations between storage time and the rate of food waste for food categories

Food Waste - Storage Time						
**Correlations**						
			Food Waste Rate - Fruit	Food Waste Rate - Vegetables	Food Waste Rate - Meat	Food Waste Rate - Fish
Spearman-Rho	Storage Time - Fruit	Correleation Coefficient	-0.015	[redacted]	[redacted]	[redacted]

		sig. (2-sided)	0.456	[redacted]	[redacted]	[redacted]
		N	2528	[redacted]	[redacted]	[redacted]
	Storage Time - Vegetables	Correleation Coefficient	[redacted]	-,103**	[redacted]	[redacted]
		sig. (2-sided)	[redacted]	0.000	[redacted]	[redacted]
		N	[redacted]	2584	[redacted]	[redacted]
	Storage Time - Meat	Correleation Coefficient	[redacted]	[redacted]	-,168**	[redacted]
		sig. (2-sided)	[redacted]	[redacted]	0.000	[redacted]
		N	[redacted]	[redacted]	2484	[redacted]
	Storage Time - Fish	Correleation Coefficient	[redacted]	[redacted]	[redacted]	-,169**
		sig. (2-sided)	[redacted]	[redacted]	[redacted]	0.000
		N	[redacted]	[redacted]	[redacted]	2176

Table 8: : Raw data, correlations between storage time and the rate of freshness loss for food categories

Loss of Freshness - Storage Time						
**Correlations**						
			Loss of Freshness - Fruit	Loss of Freshness - Vegetables	Loss of Freshness - Meat	Loss of Freshness - Fish
Spearman-Rho	Storage Time - Fruit	Correleation Coefficient	,085**	[redacted]	[redacted]	[redacted]
		sig. (2-sided)	0.000	[redacted]	[redacted]	[redacted]
		N	2528	[redacted]	[redacted]	[redacted]
	Storage Time - Vegetables	Correleation Coefficient	[redacted]	,094**	[redacted]	[redacted]
		sig. (2-sided)	[redacted]	[redacted]	[redacted]	[redacted]
		N	[redacted]	[redacted]	[redacted]	[redacted]
	Storage Time - Meat	Correleation Coefficient	[redacted]	[redacted]	0.036	[redacted]
		sig. (2-sided)	[redacted]	[redacted]	[redacted]	[redacted]
		N	[redacted]	[redacted]	[redacted]	[redacted]
	Storage Time - Fish	Correleation Coefficient	[redacted]	[redacted]	[redacted]	-0.005
		sig. (2-sided)	[redacted]	[redacted]	[redacted]	0.813
		N	[redacted]	[redacted]	[redacted]	2176

Table 9: Raw data, correlations between the rates of food waste and freshness loss

Loss of Freshness - Food Waste						
**Correlations**						
			Loss of Freshness - Fruit	Loss of Freshness - Vegetables	Loss of Freshness - Meat	Loss of Freshness - Fish
Spearman-Rho	Food Waste Rate - Fruit	Correleation Coefficient	-,267**	[redacted]	[redacted]	[redacted]
		sig. (2-sided)	0.000	[redacted]	[redacted]	[redacted]
		N	2528	[redacted]	[redacted]	[redacted]
	Food Waste Rate - Vegetables	Correleation Coefficient	[redacted]	-,299**	[redacted]	[redacted]
		sig. (2-sided)	[redacted]	[redacted]	[redacted]	[redacted]
		N	[redacted]	[redacted]	[redacted]	[redacted]
	Food Waste Rate - Meat	Correleation Coefficient	[redacted]	[redacted]	-,258**	[redacted]

		sig. (2-sided)	[redacted]	[redacted]	0.000	[redacted]
		N	[redacted]	[redacted]	2484	[redacted]
	Food Waste Rate - Fish	Correleation Coefficient	[redacted]	[redacted]	[redacted]	-,246**
		sig. (2-sided)	[redacted]	[redacted]	[redacted]	0.000
		N	[redacted]	[redacted]	[redacted]	2176

Table 10: Raw data, correlations between the importance of food category related storage features and the rate of food waste

Food Waste Rate - Importance of Storage Features						
**Correlations**						
			Food Waste Rate - Fruit	Food Waste Rate - Vegetables	Food Waste Rate - Meat	Food Waste Rate - Fish
Spearman-Rho	Hygienic function	Correleation Coefficient	,139**	,134**	,154**	,164**
		sig. (2-sided)	0.000	0.000	0.000	0.000
		N	2528	2584	2484	2176
	Filtration of airborne micro organisms	Correleation Coefficient	,087**	,084**	,086**	0.040
		sig. (2-sided)	0.001	0.001	0.001	0.129
		N	1595	1617	1571	1413
	Temperature-controlled Compartment	Correleation Coefficient	,127**	,121**	,097**	,150**
		sig. (2-sided)	0.000	0.000	0.000	0.000
		N	2528	2584	2484	2176
	Humidity-controlled Compartment	Correleation Coefficient	[redacted]	,069**	[redacted]	[redacted]
		sig. (2-sided)	[redacted]	0.003	[redacted]	[redacted]
		N	[redacted]	1820	[redacted]	[redacted]

Table 11: Test for Between-Subject effects for household size and age groups, regarding food waste for food categories

**FRUIT**					
**Tests of Between-Subjects effects**					
dependent Variable: cx8r1: Fruit - how often do you throw fruit away because it is no longer edible?					
Source	Type III Sum of Squares	df	Mean Square	F	Sig.
Corrected Model	300.885	24	12.537	10.057	0.000
Intercept	4079.697	1	4079.697	3272.571	0.000
Household size	29.803	4	7.451	5.977	0.000
Age group	34.860	4	8.715	6.991	0.000
Household size*age group	26.587	16	1.662	1.333	0.167
Error	3120.324	2503	1.247		
Total	34980.000	2528			
Corrected Total	3421.209	2527			
**VEGETABLES**					
**Tests of Between-Subjects effects**					
dependent Variable: cx8r2: Vegetables - how often do you throw vegetables away because it is no longer edible?					

Source	Type III Sum of Squares	df	Mean Square	F	Sig.
Corrected Model	342.759	24	14.282	11.461	0.000
Intercept	4614.806	1	4614.806	3703.248	0.000
Household size	23.895	4	5.974	4.794	0.001
Age group	46.802	4	11.701	9.389	0.000
Household size*age group	25.151	16	1.572	1.261	0.213
Error	3188.900	2559	1.246		
Total	37176.000	2584			
Corrected Total	3531.659	2583			

MEAT

Tests of Between-Subjects effects

dependent Variable: cx8r4: Meat - how often do you throw meat away because it is no longer edible?

Source	Type III Sum of Squares	df	Mean Square	F	Sig.
Corrected Model	228.29	24	9.512	8.285	0.000
Intercept	5667.816	1	5667.816	4936.668	0.000
Household size	19.260	4	4.815	4.194	0.002
Age group	29.702	4	7.425	6.468	0.000
Household size*age group	15.483	16	0.968	0.843	0.637
Error	2823.192	2459	1.148		
Total	45794.000	2484			
Corrected Total	3051.481	2483			

FISH

Tests of Between-Subjects effects

dependent Variable: cx8r3: Fish - how often do you throw fish away because it is no longer edible?

Source	Type III Sum of Squares	df	Mean Square	F	Sig.
Corrected Model	153.883	24	6.412	6.354	0.000
Intercept	5962.445	1	5962.445	5908.719	0.000
Household size	18.223	4	4.556	4.515	0.001
Age group	16.004	4	4.001	3.965	0.003
Household size*age group	18.994	16	1.187	1.176	0.279
Error	2170.558	2151	1.009		
Total	43208.000	2176			
Corrected Total	2324.441	2175			

Table 12: Test for Between-Subject effects for household size and storage duration, regarding food waste for food categories

FRUIT

Tests of Between-Subjects effects

dependent Variable: cx8r1: Fruit - how often do you throw fruit away because it is no longer edible?

Source	Type III Sum of Squares	df	Mean Square	F	Sig.
Corrected Model	113.581	29	3.917	2.958	0.000
Intercept	2228.709	1	2228.709	1683.175	0.000
Household size	43.294	4	10.824	8.174	0.000
Storage Duration	6.527	5	1.305	0.986	0.425
Household size*storage duration	36.777	20	1.839	1.389	0.116
Error	3307.628	2498	1.324		
Total	34980.000	2528			
Corrected Total	3421.209	2527			

VEGETABLES

Tests of Between-Subjects effects

dependent Variable: cx8r2: Vegetables - how often do you throw vegetables away because it is no longer edible?

Source	Type III Sum of Squares	df	Mean Square	F	Sig.
Corrected Model	137.435	29	4.739	3.566	0.000
Intercept	3077.228	1	3077.228	2315.475	0.000
Household size	33.238	4	8.309	6.253	0.000
Storage Duration	12.383	5	2.477	1.863	0.097
Household size*storage duration	26.221	20	1.311	0.986	0.475
Error	3394.225	2554	1.329		
Total	37176.000	2584			
Corrected Total	3531.659	2583			

MEAT

Tests of Between-Subjects effects

dependent Variable: cx8r4: Meat - how often do you throw meat away because it is no longer edible?

Source	Type III Sum of Squares	df	Mean Square	F	Sig.
Corrected Model	148.079	29	5.106	4.316	0.000
Intercept	4167.397	1	4167.397	3522.347	0.000
Household size	28.942	4	7.235	6.116	0.000
Storage Duration	26.762	5	5.352	4.524	0.000
Household size*storage duration	19.139	20	0.957	0.809	0.705
Error	2903.402	2454	1.183		
Total	45794.000	2484			
Corrected Total	3051.481	2483			

FISH

Tests of Between-Subjects effects

dependent Variable: cx8r3: Fish - how often do you throw fish away because it is no longer edible?

Source	Type III Sum of Squares	df	Mean Square	F	Sig.
Corrected Model	169.59	28	6.057	6.035	0.000
Intercept	4119.112	1	4119.112	4104.103	0.000
Household size	13.412	4	3.353	3.341	0.010
Storage Duration	61.646	5	12.329	12.284	0.000
Household size*storage duration	19.635	19	1.033	1.030	0.422
Error	2154.851	2147	1.004		
Total	43208.000	2176			
Corrected Total	2324.441	2175			

Table 13: Test for Between-Subject effects for the rate of food waste and storage duration, regarding loss of freshness for food categories

FRUIT

Tests of Between-Subjects effects

dependent Variable: cx2r1: Fruit - how often do you notiuce a loss of freshness

Source	Type III Sum of Squares	df	Mean Square	F	Sig.
Corrected Model	104,614[a]	29	3.607	10.212	0.000
Intercept	1165.068	1	1165.068	3297.985	0.000
Household size	20.116	4	5.029	14.235	0.000
Storage Duration	11.366	5	2.273	6.435	0.000
Waste Rate*Storage duration	13.350	20	0.668	1.890	0.010
Error	882.460	2498	0.353		
Total	6936.000	2528			
Corrected Total	987.074	2527			

VEGETABLES

Tests of Between-Subjects effects

dependent Variable: cx2r2: Vegetables - how often do you notiuce a loss of freshness

Source	Type III Sum of Squares	df	Mean Square	F	Sig.
Corrected Model	120,788[a]	29	4.165	11.894	0.000
Intercept	907.269	1	907.269	2590.908	0.000
Household size	24.351	4	6.088	17.385	0.000
Storage Duration	8.102	5	1.620	4.627	0.000
Waste Rate*Storage duration	13.817	20	0.691	1.973	0.006
Error	894.345	2554	0.350		
Total	7432.000	2584			
Corrected Total	1015.133	2583			

MEAT

Tests of Between-Subjects effects					
dependent Variable: cx2r4: Meat - how often do you notiuce a loss of freshness					
Source	Type III Sum of Squares	df	Mean Square	F	Sig.
Corrected Model	76,679[a]	28	2.739	9.111	0.000
Intercept	707.933	1	707.933	2355.320	0.000
Household size	21.507	4	5.377	17.889	0.000
Storage Duration	4.224	5	0.845	2.810	0.015
Waste Rate*Storage duration	7.084	19	0.373	1.240	0.214
Error	737.894	2455	0.301		
Total	5814.000	2484			
Corrected Total	814.573	2483			

FISH

Tests of Between-Subjects effects					
dependent Variable: cx2r3: Fish - how often do you notiuce a loss of freshness					
Source	Type III Sum of Squares	df	Mean Square	F	Sig.
Corrected Model	71,728[a]	29	2.473	7.846	0.000
Intercept	449.964	1	449.964	1427.288	0.000
Household size	18.989	4	4.747	15.058	0.000
Storage Duration	2.422	5	0.484	1.536	0.175
Waste Rate*Storage duration	6.335	20	0.317	1.005	0.453
Error	676.543	2146	0.315		
Total	5105.000	2176			
Corrected Total	748.272	2175			

Table 14: Raw data, linear regression for demographic characteristics (age groups) and the rate of food waste for food categories

Food Waste - Agegroup

Linear Regression Food Waste Fruit - Agegroup

Variables Entered/Removed[a]

Model	Entered variables	Removed Variables	Method
1	Agegroup[b]		enter

a. dependent variable: FoodWaste_Fruit

b. Alle gewünschten Variablen wurden eingegeben.

Model Summary

Model	R	R-Quadrat	Adjusted R Quare	Std. Error of the Estimate
1	,262[a]	0.069	0.068	1.123

a. Einflußvariablen : (Konstante), Agegroup

ANOVA[a]

Model		Sum of Squares	df	Mean Square	F	Sig.
1	Regression	234.487	1	234.487	185.869	,000[b]
	Residual	3186.722	2526	1.262		
	Total	3421.209	2527			

a. dependent variable: FoodWaste_Fruit

b. Einflußvariablen : (Konstante), Agegroup

Coefficients[a]

Model		Unstandarized Coefficients B	Unstandarized Coefficients Std.-Error	Standarized Coefficients Beta	T	Sig.
1	(Constant)	2.630	0.070		37.597	0.000
	Agegroup	0.226	0.017	0.262	13.633	0.000

a. dependent variable: FoodWaste_Fruit

Linear Regression Food Waste Vegetables - Agegroup

Variables Entered/Removed[a]

Model	Entered variables	Removed Variables	Method
1	Agegroup[b]		enter

a. dependent variable: FoodWaste_Vegetables

b. Alle gewünschten Variablen wurden eingegeben.

Model Summary

Model	R	R-Quadrat	Adjusted R Quare	Std. Error of the Estimate
1	,283[a]	0.080	0.080	1.122

a. Einflußvariablen : (Konstante), Agegroup

ANOVA[a]

Model		Sum of Squares	df	Mean Square	F	Sig.
1	Regression	282.784	1	282.784	224.739	,000[b]
	Residual	3248.876	2582	1.258		
	Total	3531.659	2583			

a. dependent variable: FoodWaste_Vegetables

b. Einflußvariablen : (Konstante), Agegroup

Coefficients[a]

Model		Unstandarized Coefficients B	Unstandarized Coefficients Std.-Error	Standarized Coefficients Beta	T	Sig.
1	(Constant)	2.625	0.069		37.913	0.000
	Agegroup	0.245	0.016	0.283	14.991	0.000

a. dependent variable: FoodWaste_Vegetables

Linear Regression Food Waste Meat - Agegroup

Variables Entered/Removed[a]

Model	Entered variables	Removed Variables	Method
1	Agegroup[b]		enter

a. dependent variable: FoodWaste_Meat

b. Alle gewünschten Variablen wurden eingegeben.

Model Summary

Model	R	R-Quadrat	Adjusted R Quare	Std. Error of the Estimate
1	,246[a]	0.061	0.060	1.075

a. Einflußvariablen : (Konstante), Agegroup

ANOVA[a]

Model		Sum of Squares	df	Mean Square	F	Sig.
1	Regression	185.137	1	185.137	160.312	,000[b]
	Residual	2866.345	2482	1.155		
	Total	3051.481	2483			

a. dependent variable: FoodWaste_Meat

b. Einflußvariablen : (Konstante), Agegroup

Coefficients[a]

Model		Unstandarized Coefficients		Standarized Coefficients		
		B	Std.-Error	Beta	T	Sig.
1	(Constant)	3.331	0.068		48.951	0.000
	Agegroup	0.203	0.016	0.246	12.661	0.000

a. dependent variable: FoodWaste_Meat

Linear Regression Food Waste Fish - Agegroup

Variables Entered/Removed[a]

Model	Entered variables	Removed Variables	Method
1	Agegroup[b]		enter

a. dependent variable: FoodWaste_Fish

b. Alle gewünschten Variablen wurden eingegeben.

Model Summary

Model	R	R-Quadrat	Adjusted R Quare	Std. Error of the Estimate
1	,220[a]	0.048	0.048	1.009

a. Einflußvariablen : (Konstante), Agegroup

ANOVA[a]

Model		Sum of Squares	df	Mean Square	F	Sig.
1	Regression	112.338	1	112.338	110.403	,000[b]
	Residual	2212.103	2174	1.018		
	Total	2324.441	2175			

a. dependent variable: FoodWaste_Fish

b. Einflußvariablen : (Konstante), Agegroup

Coefficients[a]

		Unstandarized Coefficients		Standarized Coefficients		

Model		B	Std.-Error	Beta	T	Sig.
1	(Constant)	3.655	0.068		53.581	0.000
	Agegroup	0.169	0.016	0.220	10.507	0.000

a. dependent variable: FoodWaste_Fish

Table 15: Raw data, linear regression for demographic characteristics (household size) and the rate of food waste for food categories

Linear Regression Food Waste Fruit - Household Size

Variables Entered/Removed[a]

Model	Entered variables	Removed Variables	Method
1	Household_Size[b]		Enter

a. dependent variable: FoodWaste_Fruit

b. Alle gewünschten Variablen wurden eingegeben.

Model Summary

Model	R	R-Quadrat	Adjusted R Quare	Std. Error of the Estimate
1	,142[a]	0.020	0.020	1.152

a. Einflußvariablen : (Konstante), Household_Size

ANOVA[a]

Model		Sum of Squares	df	Mean Square	F	Sig.
1	Regression	69.395	1	69.395	52.298	,000[b]
	Residual	3351.813	2526	1.327		
	Total	3421.209	2527			

a. dependent variable: FoodWaste_Fruit

b. Einflußvariablen : (Konstante), Household_Size

Coefficients[a]

Model		Unstandarized Coefficients		Standarized Coefficients		
		B	Std.-Error	Beta	T	Sig.
1	(Constant)	3.827	0.047		82.026	0.000
	Household_Size	-0.158	0.022	-0.142	-7.232	0.000

a. dependent variable: FoodWaste_Fruit

Linear Regression Food Waste Vegetables - Household Size

Variables Entered/Removed[a]

Model	Entered variables	Removed Variables	Method
1	Household_Size[b]		Enter

a. dependent variable: FoodWaste_Vegetables

b. Alle gewünschten Variablen wurden eingegeben.

Model Summary

Model	R	R-Quadrat	Adjusted R Quare	Std. Error of the Estimate
1	,137[a]	0.019	0.018	1.159

a. Einflußvariablen : (Konstante), Household_Size

ANOVA[a]

Model		Sum of Squares	df	Mean Square	F	Sig.
1	Regression	66.192	1	66.192	49.317	,000[b]
	Residual	3465.468	2582	1.342		
	Total	3531.659	2583			

a. dependent variable: FoodWaste_Vegetables

b. Einflußvariablen : (Konstante), Household_Size

Coefficients[a]

Model		Unstandarized Coefficients		Standarized Coefficients	T	Sig.
		B	Std.-Error	Beta		
1	(Constant)	3.893	0.046		83.784	0.000
	Household_Size	-0.152	0.022	-0.137	-7.023	0.000

a. dependent variable: FoodWaste_Vegetables

Linear Regression Food Waste Meat - Household Size

Variables Entered/Removed[a]

Model	Entered variables	Removed Variables	Method
1	Household_Size[b]		enter

a. dependent variable: FoodWaste_Meat

b. Alle gewünschten Variablen wurden eingegeben.

Model Summary

Model	R	R-Quadrat	Adjusted R Quare	Std. Error of the Estimate
1	,116[a]	0.013	0.013	1.101

a. Einflußvariablen : (Konstante), Household_Size

ANOVA[a]

Model		Sum of Squares	df	Mean Square	F	Sig.
1	Regression	41.100	1	41.100	33.886	,000[b]
	Residual	3010.381	2482	1.213		
	Total	3051.481	2483			

a. dependent variable: FoodWaste_Meat

b. Einflußvariablen : (Konstante), Household_Size

Coefficients[a]

Model		Unstandarized Coefficients		Standarized Coefficients	T	Sig.
		B	Std.-Error	Beta		
1	(Constant)	4.376	0.045		97.274	0.000
	Household_Size	-0.122	0.021	-0.116	-5.821	0.000

a. dependent variable: FoodWaste_Meat

Linear Regression Food Waste Fish - Household Size			

Variables Entered/Removed[a]

Model	Entered variables	Removed Variables	Method
1	Household_Size[b]		enter

a. dependent variable: FoodWaste_Fish

b. Alle gewünschten Variablen wurden eingegeben.

Model Summary

Model	R	R-Quadrat	Adjusted R Quare	Std. Error of the Estimate
1	,123[a]	0.015	0.015	1.026

a. Einflußvariablen : (Konstante), Household_Size

ANOVA[a]

Model		Sum of Squares	df	Mean Square	F	Sig.
1	Regression	35.233	1	35.233	33.460	,000[b]
	Residual	2289.208	2174	1.053		
	Total	2324.441	2175			

a. dependent variable: FoodWaste_Fish

b. Einflußvariablen : (Konstante), Household_Size

Coefficients[a]

Model		Unstandarized Coefficients		Standarized Coefficients		
		B	Std.-Error	Beta	T	Sig.
1	(Constant)	4.561	0.045		101.533	0.000
	Household_Size	-0.121	0.021	-0.123	-5.784	0.000

a. dependent variable: FoodWaste_Fish

Table 16: Raw data, linear regression for storage duration and the rate of food waste for food categories

Linear Regression Food Waste Fruit - Storage duration fruit			

Variables Entered/Removed[a]

Model	Entered variables	Removed Variables	Method
1	Storage_duration_fruit[b]		enter

a. dependent variable: FoodWaste_Fruit

b. Alle gewünschten Variablen wurden eingegeben.

Model Summary

Model	R	R-Quadrat	Adjusted R Quare	Std. Error of the Estimate
1	,005[a]	0.000	0.000	1.164

a. Einflußvariablen : (Konstante), Storage_duration_fruit

ANOVA[a]

Model		Sum of Squares	df	Mean Square	F	Sig.

1	Regression	0.102	1	0.102	0.075	,784[b]
	Residual	3421.107	2526	1.354		
	Total	3421.209	2527			

a. dependent variable: FoodWaste_Fruit

b. Einflußvariablen : (Konstante), Storage_duration_fruit

Coefficients[a]

Model		Unstandarized Coefficients B	Std.-Error	Standarized Coefficients Beta	T	Sig.
1	(Constant)	3.514	0.074		47.433	0.000
	Storage_duration_fruit	0.006	0.024	0.005	0.274	0.784

a. dependent variable: FoodWaste_Fruit

Linear Regression Food Waste Vegetables - Storage duration vegetables

Variables Entered/Removed[a]

Model	Entered variables	Removed Variables	Method
1	Storage_duration_vegetables[b]		enter

a. dependent variable: FoodWaste_Vegetables

b. Alle gewünschten Variablen wurden eingegeben.

Model Summary

Model	R	R-Quadrat	Adjusted R Quare	Std. Error of the Estimate
1	,079[a]	0.006	0.006	1.166

a. Einflußvariablen : (Konstante), Storage_duration_vegetables

ANOVA[a]

Model		Sum of Squares	df	Mean Square	F	Sig.
1	Regression	21.783	1	21.783	16.024	,000[b]
	Residual	3509.877	2582	1.359		
	Total	3531.659	2583			

a. dependent variable: FoodWaste_Vegetables

b. Einflußvariablen : (Konstante), Storage_duration_vegetables

Coefficients[a]

Model		Unstandarized Coefficients B	Std.-Error	Standarized Coefficients Beta	T	Sig.
1	(Constant)	3.890	0.074		52.499	0.000
	Storage_duration_vegetables	-0.094	0.023	-0.079	-4.003	0.000

a. dependent variable: FoodWaste_Vegetables

Linear Regression Food Waste Meat - Storage duration meat

Variables Entered/Removed[a]

Model	Entered variables	Removed Variables	Method

1	Storage_duration_meat[b]		enter

a. dependent variable: FoodWaste_Meat

b. Alle gewünschten Variablen wurden eingegeben.

Model Summary

Model	R	R-Quadrat	Adjusted R Quare	Std. Error of the Estimate
1	,105[a]	0.011	0.011	1.103

a. Einflußvariablen : (Konstante), Storage_duration_meat

ANOVA[a]

Model		Sum of Squares	df	Mean Square	F	Sig.
1	Regression	33.922	1	33.922	27.902	,000[b]
	Residual	3017.559	2482	1.216		
	Total	3051.481	2483			

a. dependent variable: FoodWaste_Meat

b. Einflußvariablen : (Konstante), Storage_duration_meat

Coefficients[a]

Model		Unstandarized Coefficients B	Unstandarized Coefficients Std.-Error	Standarized Coefficients Beta	T	Sig.
1	(Constant)	4.425	0.057		77.737	0.000
	Storage_duration_meat	-0.111	0.021	-0.105	-5.282	0.000

a. dependent variable: FoodWaste_Meat

Linear Regression Food Waste Fish - Storage duration fish

Variables Entered/Removed[a]

Model	Entered variables	Removed Variables	Method
1	Storage_duration_fish[b]		enter

a. dependent variable: FoodWaste_Fish

b. Alle gewünschten Variablen wurden eingegeben.

Model Summary

Model	R	R-Quadrat	Adjusted R Quare	Std. Error of the Estimate
1	,115[a]	0.013	0.013	1.027

a. Einflußvariablen : (Konstante), Storage_duration_fish

ANOVA[a]

Model		Sum of Squares	df	Mean Square	F	Sig.
1	Regression	30.888	1	30.888	29.278	,000[b]
	Residual	2293.553	2174	1.055		
	Total	2324.441	2175			

a. dependent variable: FoodWaste_Fish

b. Einflußvariablen : (Konstante), Storage_duration_fish

Model		B	Std.-Error	Beta	T	Sig.
		Unstandarized Coefficients		Standarized Coefficients		
1	(Constant)	4.551	0.046		99.814	0.000
	Storage_duration_fish	-0.107	0.020	-0.115	-5.411	0.000

Coefficients[a]

a. dependent variable: FoodWaste_Fish

Table 17: Raw data, linear regression for the rate of freshness loss and the storage duration for food categories

Linear Regression Loss of Freshness Fruit - Storage duration fruit

Variables Entered/Removed[a]

Model	Entered variables	Removed Variables	Method
1	Storage_time_Fruit[b]		enter

a. dependent variable: Freshness_loss_fruit

b. Alle gewünschten Variablen wurden eingegeben.

Model Summary

Model	R	R-Quadrat	Adjusted R Quare	Std. Error of the Estimate
1	,087[a]	0.008	0.007	0.623

a. Einflußvariablen : (Konstante), Storage_time_Fruit

ANOVA[a]

Model		Sum of Squares	df	Mean Square	F	Sig.
1	Regression	7.431	1	7.431	19.162	,000[b]
	Residual	979.643	2526	0.388		
	Total	987.074	2527			

a. dependent variable: Freshness_loss_fruit

b. Einflußvariablen : (Konstante), Storage_time_Fruit

Coefficients[a]

Model		Unstandarized Coefficients B	Unstandarized Coefficients Std.-Error	Standarized Coefficients Beta	T	Sig.
1	(Constant)	1.369	0.040		34.538	0.000
	Storage_time_Fruit	0.055	0.013	0.087	4.377	0.000

a. dependent variable: Freshness_loss_fruit

Linear Regression Loss of Freshness Vegetables - Storage duration Vegetables

Variables Entered/Removed[a]

Model	Entered variables	Removed Variables	Method
1	Storage_time_Vegetables[b]		enter

a. dependent variable: Freshness_loss_vegetables

b. Alle gewünschten Variablen wurden eingegeben.

Model Summary

Model	R	R-Quadrat	Adjusted R Quare	Std. Error of the Estimate
1	,104[a]	0.011	0.010	0.624

a. Einflußvariablen : (Konstante), Storage_time_Vegetables

ANOVA[a]

Model		Sum of Squares	df	Mean Square	F	Sig.
1	Regression	11.009	1	11.009	28.309	,000[b]
	Residual	1004.124	2582	0.389		
	Total	1015.133	2583			

a. dependent variable: Freshness_loss_vegetables

b. Einflußvariablen : (Konstante), Storage_time_Vegetables

Coefficients[a]

Model		Unstandarized Coefficients		Standarized Coefficients		
		B	Std.-Error	Beta	T	Sig.
1	(Constant)	1.375	0.040		34.698	0.000
	Storage_time_ Vegetables	0.067	0.013	0.104	5.321	0.000

a. dependent variable: Freshness_loss_vegetables

Linear Regression Loss of Freshness Meat - Storage duration Meat

Variables Entered/Removed[a]

Model	Entered variables	Removed Variables	Method
1	Storage_time_ Meat[b]		enter

a. dependent variable: Freshness_loss_meat

b. Alle gewünschten Variablen wurden eingegeben.

Model Summary

Model	R	R-Quadrat	Adjusted R Quare	Std. Error of the Estimate
1	,016[a]	0.000	0.000	0.573

a. Einflußvariablen : (Konstante), Storage_time_Meat

ANOVA[a]

Model		Sum of Squares	df	Mean Square	F	Sig.
1	Regression	0.201	1	0.201	0.613	,434[b]
	Residual	814.372	2482	0.328		
	Total	814.573	2483			

a. dependent variable: Freshness_loss_meat

b. Einflußvariablen : (Konstante), Storage_time_Meat

Coefficients[a]

Model		Unstandarized Coefficients		Standarized Coefficients		
		B	Std.-Error	Beta	T	Sig.
1	(Constant)	1.397	0.030		47.251	0.000

	Storage_time_Meat	0.009	0.011	0.016	0.783	0.434

a. dependent variable: Freshness_loss_meat

Linear Regression Loss of Freshness Fish - Storage duration Fish

Variables Entered/Removed[a]

Model	Entered variables	Removed Variables	Method
1	Storage_time_Fish[b]		enter

a. dependent variable: Freshness_loss_fish

b. Alle gewünschten Variablen wurden eingegeben.

Model Summary

Model	R	R-Quadrat	Adjusted R Quare	Std. Error of the Estimate
1	,019[a]	0.000	0.000	0.587

a. Einflußvariablen : (Konstante), Storage_time_Fish

ANOVA[a]

Model		Sum of Squares	df	Mean Square	F	Sig.
1	Regression	0.268	1	0.268	0.778	,378[b]
	Residual	748.004	2174	0.344		
	Total	748.272	2175			

a. dependent variable: Freshness_loss_fish

b. Einflußvariablen : (Konstante), Storage_time_Fish

Coefficients[a]

Model		Unstandarized Coefficients		Standarized Coefficients	T	Sig.
		B	Std.-Error	Beta		
1	(Constant)	1.435	0.026		55.120	0.000
	Storage_time_Fish	-0.010	0.011	-0.019	-0.882	0.378

a. dependent variable: Freshness_loss_fish

Table 18: Raw data, linear regression for the rate of food waste for food categories and the importance of storage features

Food Waste - Importance of Storage Features

Linear Regression Food Waste Fruit - Importance of Hygienic Function

Variables Entered/Removed[a]

Model	Entered variables	Removed Variables	Method
1	Importance_Hygienic_Function[b]		enter

a. dependent variable: Food_Waste_Fruit

b. Alle gewünschten Variablen wurden eingegeben.

Model Summary

Model	R	R-Quadrat	Adjusted R Quare	Std. Error of the Estimate

1	,000[a]	0.000	0.000	1.164

a. Einflußvariablen : (Konstante), Importance_Hygienic_Function

ANOVA[a]

Model		Sum of Squares	df	Mean Square	F	Sig.
1	Regression	0.000	1	0.000	0.000	,998[b]
	Residual	3421.209	2526	1.354		
	Total	3421.209	2527			

a. dependent variable: Food_Waste_Fruit

b. Einflußvariablen : (Konstante), Importance_Hygienic_Function

Coefficients[a]

Model		Unstandarized Coefficients B	Std.-Error	Standarized Coefficients Beta	T	Sig.
1	(Constant)	3.533	0.057		61.549	0.000
	Importance_Hygienic_Function	7.429E-05	0.033	0.000	0.002	0.998

a. dependent variable: Food_Waste_Fruit

Linear Regression Food Waste Fruit - Importance of Airfiltration

Variables Entered/Removed[a]

Model	Entered variables	Removed Variables	Method
1	Importance_Airfiltration[b]		enter

a. dependent variable: Food_Waste_Fruit

b. Alle gewünschten Variablen wurden eingegeben.

Model Summary

Model	R	R-Quadrat	Adjusted R Quare	Std. Error of the Estimate
1	,027[a]	0.001	0.000	1.162

a. Einflußvariablen : (Konstante), Importance_Airfiltration

ANOVA[a]

Model		Sum of Squares	df	Mean Square	F	Sig.
1	Regression	2.505	1	2.505	1.853	,174[b]
	Residual	3402.628	2518	1.351		
	Total	3405.133	2519			

a. dependent variable: Food_Waste_Fruit

b. Einflußvariablen : (Konstante), Importance_Airfiltration

Coefficients[a]

Model		Unstandarized Coefficients B	Std.-Error	Standarized Coefficients Beta	T	Sig.
1	(Constant)	3.608	0.060		60.582	0.000
	Importance_Airfiltration	-0.044	0.032	-0.027	-1.361	0.174

a. dependent variable: Food_Waste_Fruit

Linear Regression Food Waste Fruit - Importance of Temperature control

Variables Entered/Removed[a]

Model	Entered variables	Removed Variables	Method
1	Importance_Temperature_Control[b]		enter

a. dependent variable: Food_Waste_Fruit

b. Alle gewünschten Variablen wurden eingegeben.

Model Summary

Model	R	R-Quadrat	Adjusted R Quare	Std. Error of the Estimate
1	,004[a]	0.000	0.000	1.163

a. Einflußvariablen : (Konstante), Importance_Temperature_Control

ANOVA[a]

Model		Sum of Squares	df	Mean Square	F	Sig.
1	Regression	0.047	1	0.047	0.035	,851[b]
	Residual	3337.518	2467	1.353		
	Total	3337.565	2468			

a. dependent variable: Food_Waste_Fruit

b. Einflußvariablen : (Konstante), Importance_Temperature_Control

Coefficients[a]

Model		Unstandarized Coefficients		Standarized Coefficients	T	Sig.
		B	Std.-Error	Beta		
1	(Constant)	3.516	0.059		59.295	0.000
	Importance_Temperature_Control	0.006	0.031	0.004	0.187	0.851

a. dependent variable: Food_Waste_Fruit

Linear Regression Food Waste Vegetables - Importance of Hygienic Function

Variables Entered/Removed[a]

Model	Entered variables	Removed Variables	Method
1	Importance_Hygienic_Function[b]		enter

a. dependent variable: Food_Waste_Vegetables

b. Alle gewünschten Variablen wurden eingegeben.

Model Summary

Model	R	R-Quadrat	Adjusted R Quare	Std. Error of the Estimate
1	,002[a]	0.000	0.000	1.171

a. Einflußvariablen : (Konstante), Importance_Hygienic_Function

ANOVA[a]

Model		Sum of Squares	df	Mean Square	F	Sig.
1	Regression	0.015	1	0.015	0.011	,918[b]
	Residual	3489.887	2545	1.371		
	Total	3489.902	2546			

a. dependent variable: Food_Waste_Vegetables

b. Einflußvariablen : (Konstante), Importance_Hygienic_Function

Coefficients[a]

Model		Unstandarized Coefficients		Standarized Coefficients	T	Sig.
		B	Std.-Error	Beta		
1	(Constant)	3.608	0.058		62.579	0.000
	Importance_Hygienic_Function	-0.003	0.033	-0.002	-0.103	0.918

a. dependent variable: Food_Waste_Vegetables

Linear Regression Food Waste Vegetables - Importance of Airfiltration

Variables Entered/Removed[a]

Model	Entered variables	Removed Variables	Method
1	Importance_Airfiltration[b]		enter

a. dependent variable: Food_Waste_Vegetables

b. Alle gewünschten Variablen wurden eingegeben.

Model Summary

Model	R	R-Quadrat	Adjusted R Quare	Std. Error of the Estimate
1	,062[a]	0.004	0.003	1.167

a. Einflußvariablen : (Konstante), Importance_Airfiltration

ANOVA[a]

Model		Sum of Squares	df	Mean Square	F	Sig.
1	Regression	13.693	1	13.693	10.050	,002[b]
	Residual	3517.967	2582	1.362		
	Total	3531.659	2583			

a. dependent variable: Food_Waste_Vegetables

b. Einflußvariablen : (Konstante), Importance_Airfiltration

Coefficients[a]

Model		Unstandarized Coefficients		Standarized Coefficients	T	Sig.
		B	Std.-Error	Beta		
1	(Constant)	3.781	0.059		63.963	0.000
	Importance_Airfiltration	-0.101	0.032	-0.062	-3.170	0.002

a. dependent variable: Food_Waste_Vegetables

Linear Regression Food Waste Vegetables - Temperature Control

Variables Entered/Removed[a]

Model	Entered variables	Removed Variables	Method
1	Importance_Temperature_Control[b]		enter

a. dependent variable: Food_Waste_Vegetables
b. Alle gewünschten Variablen wurden eingegeben.

Model Summary

Model	R	R-Quadrat	Adjusted R Quare	Std. Error of the Estimate
1	,004[a]	0.000	0.000	1.170

a. Einflußvariablen : (Konstante), Importance_Temperature_Control

ANOVA[a]

Model		Sum of Squares	df	Mean Square	F	Sig.
1	Regression	0.062	1	0.062	0.045	,832[b]
	Residual	3452.566	2522	1.369		
	Total	3452.628	2523			

a. dependent variable: Food_Waste_Vegetables
b. Einflußvariablen : (Konstante), Importance_Temperature_Control

Coefficients[a]

Model		Unstandarized Coefficients B	Unstandarized Coefficients Std.-Error	Standardized Coefficients Beta	T	Sig.
1	(Constant)	3.614	0.059		61.337	0.000
	Importance_Temperature_Control	-0.007	0.031	-0.004	-0.212	0.832

a. dependent variable: Food_Waste_Vegetables

Linear Regression Food Waste Vegetables - Humidity Control

Variables Entered/Removed[a]

Model	Entered variables	Removed Variables	Method
1	Importance_Humidity_Control[b]		enter

a. dependent variable: Food_Waste_Vegetables
b. Alle gewünschten Variablen wurden eingegeben.

Model Summary

Model	R	R-Quadrat	Adjusted R Quare	Std. Error of the Estimate
1	,006[a]	0.000	0.000	1.177

a. Einflußvariablen : (Konstante), Importance_Humidity_Control

ANOVA[a]

Model		Sum of Squares	df	Mean Square	F	Sig.

1	Regression	0.121	1	0.121	0.088	,767[b]
	Residual	3333.061	2405	1.386		
	Total	3333.182	2406			

a. dependent variable: Food_Waste_Vegetables

b. Einflußvariablen : (Konstante), Importance_Humidity_Control

Coefficients[a]

Model		Unstandarized Coefficients B	Unstandarized Coefficients Std.-Error	Standarized Coefficients Beta	T	Sig.
1	(Constant)	3.576	0.061		58.362	0.000
	Importance_Humidity_Control	0.010	0.033	0.006	0.296	0.767

a. dependent variable: Food_Waste_Vegetables

Linear Regression Food Waste Meat - Importance of Hygienic Function

Variables Entered/Removed[a]

Model	Entered variables	Removed Variables	Method
1	Importance_Hygienic_Function[b]		enter

a. dependent variable: Food_Waste_Meat

b. Alle gewünschten Variablen wurden eingegeben.

Model Summary

Model	R	R-Quadrat	Adjusted R Quare	Std. Error of the Estimate
1	,055[a]	0.003	0.003	1.112

a. Einflußvariablen : (Konstante), Importance_Hygienic_Function

ANOVA[a]

Model		Sum of Squares	df	Mean Square	F	Sig.
1	Regression	9.062	1	9.062	7.328	,007[b]
	Residual	2997.443	2424	1.237		
	Total	3006.505	2425			

a. dependent variable: Food_Waste_Meat

b. Einflußvariablen : (Konstante), Importance_Hygienic_Function

Coefficients[a]

Model		Unstandarized Coefficients B	Unstandarized Coefficients Std.-Error	Standarized Coefficients Beta	T	Sig.
1	(Constant)	4.002	0.056		71.254	0.000
	Importance_Hygienic_Function	0.087	0.032	0.055	2.707	0.007

a. dependent variable: Food_Waste_Meat

Linear Regression Food Waste Meat - Importance of Airfiltration

Variables Entered/Removed[a]

Model	Entered variables	Removed Variables	Method
1	Importance_Airfiltration[b]		enter

a. dependent variable: Food_Waste_Meat

b. Alle gewünschten Variablen wurden eingegeben.

Model Summary

Model	R	R-Quadrat	Adjusted R Quare	Std. Error of the Estimate
1	,016[a]	0.000	0.000	1.110

a. Einflußvariablen : (Konstante), Importance_Airfiltration

ANOVA[a]

Model		Sum of Squares	df	Mean Square	F	Sig.
1	Regression	0.759	1	0.759	0.616	,433[b]
	Residual	3018.609	2450	1.232		
	Total	3019.369	2451			

a. dependent variable: Food_Waste_Meat

b. Einflußvariablen : (Konstante), Importance_Airfiltration

Coefficients[a]

Model		Unstandarized Coefficients		Standarized Coefficients	T	Sig.
		B	Std.-Error	Beta		
1	(Constant)	4.108	0.058		71.179	0.000
	Importance_Airfiltration	0.025	0.031	0.016	0.785	0.433

a. dependent variable: Food_Waste_Meat

Linear Regression Food Waste Meat - Importance of Temperature Control

Variables Entered/Removed[a]

Model	Entered variables	Removed Variables	Method

a. dependent variable: Food_Waste_Meat

b. Alle gewünschten Variablen wurden eingegeben.

Model Summary

Model	R	R-Quadrat	Adjusted R Quare	Std. Error of the Estimate
1	,019[a]	0.000	0.000	1.109

a. Einflußvariablen : (Konstante), Importance_Temperature_Control

ANOVA[a]

Model		Sum of Squares	df	Mean Square	F	Sig.
1	Regression	1.120	1	1.120	0.911	,340[b]
	Residual	3050.362	2482	1.229		
	Total	3051.481	2483			

a. dependent variable: Food_Waste_Meat

b. Einflußvariablen : (Konstante), Importance_Temperature_Control

Coefficients[a]

Model		Unstandarized Coefficients B	Std.-Error	Standarized Coefficients Beta	T	Sig.
1	(Constant)	4.197	0.056		74.771	0.000
	Importance_Temperature_Control	-0.028	0.029	-0.019	-0.955	0.340

a. dependent variable: Food_Waste_Meat

Linear Regression Food Waste Fish - Importance of Hygienic Function

Variables Entered/Removed[a]

Model	Entered variables	Removed Variables	Method
1	Importance_Hygienic_Function[b]		enter

a. dependent variable: Food_Waste_Fish

b. Alle gewünschten Variablen wurden eingegeben.

Model Summary

Model	R	R-Quadrat	Adjusted R Quare	Std. Error of the Estimate
1	,031[a]	0.001	0.001	1.038

a. Einflußvariablen : (Konstante), Importance_Hygienic_Function

ANOVA[a]

Model		Sum of Squares	df	Mean Square	F	Sig.
1	Regression	2.255	1	2.255	2.092	,148[b]
	Residual	2310.796	2144	1.078		
	Total	2313.051	2145			

a. dependent variable: Food_Waste_Fish

b. Einflußvariablen : (Konstante), Importance_Hygienic_Function

Coefficients[a]

Model		Unstandarized Coefficients B	Std.-Error	Standarized Coefficients Beta	T	Sig.
1	(Constant)	4.255	0.056		76.648	0.000
	Importance_Hygienic_Function	0.046	0.032	0.031	1.447	0.148

a. dependent variable: Food_Waste_Fish

Linear Regression Food Waste Fish - Importance of Temperature Control

Variables Entered/Removed[a]

Model	Entered variables	Removed Variables	Method
1	Importance_Airfiltration[b]		enter

a. dependent variable: Food_Waste_Fish

b. Alle gewünschten Variablen wurden eingegeben.

Model Summary

Model	R	R-Quadrat	Adjusted R Quare	Std. Error of the Estimate
1	,007[a]	0.000	0.000	1.036

a. Einflußvariablen : (Konstante), Importance_Airfiltration

ANOVA[a]

Model		Sum of Squares	df	Mean Square	F	Sig.
1	Regression	0.122	1	0.122	0.114	,736[b]
	Residual	2321.211	2164	1.073		
	Total	2321.333	2165			

a. dependent variable: Food_Waste_Fish

b. Einflußvariablen : (Konstante), Importance_Airfiltration

Coefficients[a]

Model		Unstandarized Coefficients		Standarized Coefficients		
		B	Std.-Error	Beta	T	Sig.
1	(Constant)	4.351	0.057		75.736	0.000
	Importance_Airfiltration	-0.010	0.031	-0.007	-0.337	0.736

a. dependent variable: Food_Waste_Fish

Linear Regression Food Waste Fish - Importance of Temperature Control

Variables Entered/Removed[a]

Model	Entered variables	Removed Variables	Method
1	Importance_Temperature_Control[b]		enter

a. dependent variable: Food_Waste_Fish

b. Alle gewünschten Variablen wurden eingegeben.

Model Summary

Model	R	R-Quadrat	Adjusted R Quare	Std. Error of the Estimate
1	,027[a]	0.001	0.000	1.036

a. Einflußvariablen : (Konstante), Importance_Temperature_Control

ANOVA[a]

Model		Sum of Squares	df	Mean Square	F	Sig.
1	Regression	1.705	1	1.705	1.588	,208[b]
	Residual	2314.735	2156	1.074		
	Total	2316.439	2157			

a. dependent variable: Food_Waste_Fish

b. Einflußvariablen : (Konstante), Importance_Temperature_Control

Coefficients[a]

Model		Unstandarized Coefficients		Standarized Coefficients		
		B	Std.-Error	Beta	T	Sig.

1	(Constant)	4.266	0.056		75.561	0.000
	Importance_Temperature_Control	0.037	0.030	0.027	1.260	0.208

a. dependent variable: Food_Waste_Fish
